# The intriguing evolutionary dynamics of plant mitochondrial DNA

**DOI:** 10.1186/1741-7007-9-61

**Published:** 2011-09-27

**Authors:** Nicolas Galtier

**Affiliations:** 1 Université Montpellier 2, CNRS UMR 5554-Institut des Sciences de l'Evolution, Place E Bataillon-CC64, 34095 Montpellier, France

## Abstract

The mitochondrial genome of plants is-in every respect and for yet unclear reasons-very different from the well-studied one of animals. Thanks to next-generation sequencing technologies, Davila *et al*. precisely characterized the role played by recombination and DNA repair in controlling mitochondrial variations in *Arabidopsis thaliana*, thus opening new perspectives on the long-term evolution of this intriguing genome.

See research article: http://www.biomedcentral.com/1741-7007/9/64

The mitochondrial genome of plants is a challenge to molecular evolutionary biologists. Its content is highly dynamic: plant mitochondrial DNA (mtDNA) is large and variable in size (200 to 2,500 kb), contains many introns and repeated elements (typically 90% of the total sequence), and experiences frequent gene gain/loss/transfer/duplication, and genome rearrangements [1]. Its nucleotide substitution rate, paradoxically, is remarkably low-even lower than for nuclear DNA. These features are in sharp contrast with the highly studied mtDNA of animals, which is small-sized, structurally conserved, devoid of selfish elements, and has a very fast nucleotide substitution rate [2]. Why these two genomes behave so differently is one of the most head-scratching questions of current comparative genomics. The study by Davila *et al*. [3] contributes a potentially decisive argument by connecting the plant mtDNA mutation rate to yet another intriguing feature of this organellar genome-recombination.

## DNA repair: keeping a watch on repeated elements

Unlike in animal mitochondria, recombination is widespread in plant mtDNA. Recombinant molecules have been detected in various species, and are involved in a number of phenotypic traits, including thermotolerance and male sterility. The nuclear-encoded *MSH1 *gene, which is the product of fusion between a homologue of the bacterial DNA-repair gene *MutS *and an endonuclease gene, is involved in the control of plant mtDNA recombination. Using next-generation sequencing technologies, Davila *et al*. [3] have examined the recombination pattern in wild-type versus *MSH1*-mutant ecotypes of the Brassicaceae *Arabidopsis thaliana *model. They report that *MSH1 *mutants experience mitochondrial recombination at a much higher rate than the wild type, as reflected by the frequent detection of rearranged mitochondrial molecules generated by illegitimate (ectopic) recombination between repeated elements. Interestingly, recombination in *MSH1 *mutants is shown to be associated with asymmetrical genetic exchanges: in a window of a few hundred bases surrounding the recombination breakpoint, one of the two recombining DNA sequences is copied and pasted onto the other one. This process, known as gene conversion, is mediated by efficient DNA mismatch repair activity, and contributes to sequence homogenization of recombinogenic motifs. An analysis of mitochondrial variations across 72 natural ecotypes of *A. thaliana *reveals similar patterns, suggesting that the processes described by Davila *et al*. actually impact on the evolution of plant mtDNA.

This study, therefore, provides us with a proximal explanation for the low substitution rate of plant mtDNA, namely the existence of efficient recombination-associated DNA repair activity. Ectopic recombination is potentially harmful in generating chromosomal rearrangements that disrupt coding frames or impede gene expression regulation. Mechanisms of recombination surveillance and repair of recombination-induced DNA damage, including mismatch repair, appear necessary for plant mtDNA. Animal mtDNA, in contrast, is essentially devoid of repeated elements, so illegitimate recombination is much less an issue in these genomes. Selective pressure for efficient DNA repair might thus be relaxed, leading to an increased mutation rate. We note that, in turn, its elevated mutation rate has been invoked to explain the absence of introns in animal mtDNA-in a highly mutable context, the functional sequences necessary to proper intron excision imply an additional, counter-selected mutation load [4]. The contrast between plant and animal mtDNA behaviour might therefore reflect the two distinct solutions they implement to cope with repeated element threats: either avoiding them, at the cost of a high point mutation rate (animals), or repairing the damage they cause by selecting for efficient DNA repair activity (plants). This response, however, gives rise to another question: what properties of plants and animals, if any, have made them take such distinctive pathways regarding mitochondrial evolution?

## Lesson from corrals and sponges

An exciting clue comes from the report of plant-like mtDNA evolution in two specific lineages of animals. Like plants, the mitochondrial genomes of Porifera (sponges) and Anthozoa (corrals and sea anemones) include introns, intergenic spacers, genes of foreign origin, and selfish elements, and show an extremely low substitution rate [5]. Remarkably, a gene similar in structure to the plant *MSH1 *gene-a fusion between a *MutS *homologue and an endonuclease gene-convergently evolved in the soft corral *Sarcophyton glaucum *[6], thus giving even more credit to the link established by Davila *et al*. [3] between recombination, genome dynamics and substitution rate. Porifera and Anthozoa share, with plants, an important feature in their life cycle: a developmentally late distinction between somatic and germline cells. This feature might be of primary importance in mtDNA dynamics and evolution, as we shall now see.

In mammals, as in most animals, germ cell differentiation occurs early in embryonic development. During the series of mitoses leading from fertilized oocytes to primordial germ cells, mitochondrial replication is downregulated, and the per-cell number of mitochondria drops from hundreds of thousands to approximately ten [7]. This germline bottleneck explains the rapid segregation of mitochondrial variants across generations. It is thought to have evolved to decrease heteroplasmy, the co-existence of several mitochondrial haplotypes within the same individual. Avoiding heteroplasmy is beneficial for the nuclear genome since it reduces the opportunity for selfish mtDNA mutations to increase in frequency in the population [8]. Such a germline bottleneck is not documented in plants, perhaps as a consequence of their delayed differentiation of reproductive cells. Gene conversion and efficient mismatch repair in plants might therefore be interpreted as mechanisms selected in the nuclear genome to homogenize mtDNA sequences within an individual, thus limiting the spread of selfish mitochondrial variants, in the absence of a germline bottleneck. The syndrome of cytoplasmic male sterility, in which mtDNA variants arrest pollen growth, illustrates the major role played by mitochondrial heteroplasmy in the nucleo-cytoplasmic conflict in plants [9], and the potential benefit for the nucleus to control it.

## Mitochondrial mutations, life cycle and ageing

Although nucleo-cytoplasmic conflicts certainly affect mitochondrial evolutionary dynamics, they probably do not explain every aspect of the difference between plant and animal mitochondrial genomes First, we note that in *S. glaucum *(soft corral) the *MutS *homologue, similar in function to the plant *MSH1 *gene, is carried by the mitochondrial genome, not the nuclear genome. The hypothesis of nuclear control of mitochondrial heteroplasmy does not seem to hold here. Second, one could object that nucleo-cytoplasmic conflict arguments should apply to the chloroplast as well. Although recombination and DNA repair systems are documented in chloroplasts, they do not result in a particularly low nucleotide substitution rate or in a particularly high rearrangement rate in this organelle. The very low point mutation rate of mtDNA in plants, sponges and anthozoans apparently reflects a mitochondrion-specific property. In animals, mitochondria play a major role in a number of cellular and physiological functions, including respiration, lipid metabolism, apoptosis and, importantly, senescence. The mitochondrial theory of ageing stipulates that senescence occurs through the accumulation of molecular damage caused by oxidative by-products of mitochondrial respiration. It was recently proposed that the mitochondrial mutation rate might consequently be constrained to low values in long-lived animals in order to avoid premature somatic senescence [10]. This model would account for the low mtDNA nucleotide substitution rate of corrals, whose colonies can grow asexually for hundreds of years. It is unclear whether this hypothesis is relevant to plants, which like a well-defined dispensable soma, and in which a role of mitochondria in senescence and longevity has not been established.

The peculiarities of plant mtDNA are still largely unexplained. Although major advances have been made in identifying the underlying molecular mechanisms, we are only starting to consider their ultimate causes. Multi-level selection and genomic conflicts appear to be pivotal in this process, but the details are hypothetical. A promising opportunity to make progress is offered by a couple of plant genera in which particular species have recently experienced a fantastic increase in point mutation rate, reaching values similar to those typical of animals [11]. Understanding these exceptions might help explain the rule, and eventually solve the mystery, of the plant mitochondrial genome.
